# Co-injection of Bile and Indocyanine Green for Detecting Pancreaticobiliary Maljunction of Choledochal Cyst

**DOI:** 10.1055/s-0042-1747913

**Published:** 2022-08-23

**Authors:** Shun Onishi, Koji Yamada, Masakazu Murakami, Chihiro Kedoin, Mitsuru Muto, Satoshi Ieiri

**Affiliations:** 1 Department of Pediatric Surgery, Kagoshima University Graduate School of Medicine and Dental Sciences, Kagoshima, Sakuragaoka, Japan

**Keywords:** indocyanine green, pancreaticobiliary maljunction, choledochal cyst, near-infrared fluorescence imaging, laparoscopic surgery

## Abstract

The usage of near-infrared (NIR) fluorescence imaging with indocyanine green (ICG) has gained popularity in many procedures in pediatric surgery. ICG generates fluorescent light only when it combines with a protein. We herein report a novel technique for detecting pancreaticobiliary maljunction (PBMJ) with co-injection of bile and ICG in laparoscopic choledochal cyst resection and hepaticojejunostomy for a pediatric patient. A 4-year-old girl presented with abdominal pain and intermittent vomiting. Enhanced computed tomography and magnetic resonance cholangiopancreatography showed a 17-mm type Ia choledochal cyst. Definitive PBMJ was not detected preoperatively. Laparoscopic choledochal cyst resection and hepaticojejunostomy were performed using five ports. A percutaneous silicon catheter was inserted into the gallbladder, and bile juice was aspirated. The amylase level of the bile juice was over 3 × 105 IU/L. The aspirated bile juice and ICG were mixed and co-injected into the gallbladder through the catheter. ICG combined with protein in bile juice and generated fluorescent light. Dilated common bile duct and pancreas were detected by NIR fluorescence imaging. This imaging technique was helpful for detecting the dissection margin of the distal side of the choledochal cyst inside the pancreatic tissue and preventing injury of the pancreatic tissue. This is the first case of ICG application for laparoscopic choledochal cyst resection in a pediatric patient. After resection of the choledochal cyst, laparoscopic hepaticojejunostomy was completely performed. Our technique is a safe and low-invasive method of detecting and excising the distal side of the cyst without a risk of radiography and residual bile duct.

## Introduction

Previous research has confirmed that patients with choledochal cysts have an elevated risk of cholangiocarcinoma. Complete choledochal cyst excision is necessary to prevent cholangiocarcinoma. 1 The transection level of the pancreatic side of the choledochal cyst is generally determined with intraoperative cholangiography using iodine contrast agents; however, repeat cholangiography is often required to confirm the transection level, carrying a risk of radiation exposure.

The use of near-infrared (NIR) fluorescence imaging with indocyanine green (ICG) has gained popularity in many procedures in pediatric surgery. 2 The vascular anatomy, lymphatic duct, hepatocyte and biliary structure can be detected with NIR fluorescence imaging. ICG use in laparoscopic cholecystectomy for pediatric patients has been reported; 3 however, there have been no reports of its application in laparoscopic choledochal cyst excision.

Because ICG generates fluorescent light only when it combines with serum proteins, it is usually injected into a vein. 4 We herein report a novel technique for detecting pancreaticobiliary maljunction (PBMJ) and determining the transection level of choledochal cyst with co-injection of bile and ICG into the bile duct in laparoscopic choledochal cyst excision and hepaticojejunostomy for a pediatric patient.

## Preliminary Ex vivo Experiment

First, we mixed ICG and bile ex vivo and performed observation using a near-infrared/ICG camera (KARL STORZ, Tuttlingen, Germany). Bile extracted from another patient who underwent laparoscopic cholecystectomy was mixed with ICG at various concentrations. The strongest fluorescence image was obtained with a mixture of 30% ICG and 70% bile, so we planned to use this concentration for laparoscopic choledochal cyst excision.

## Case Report

**Fig. 1:**
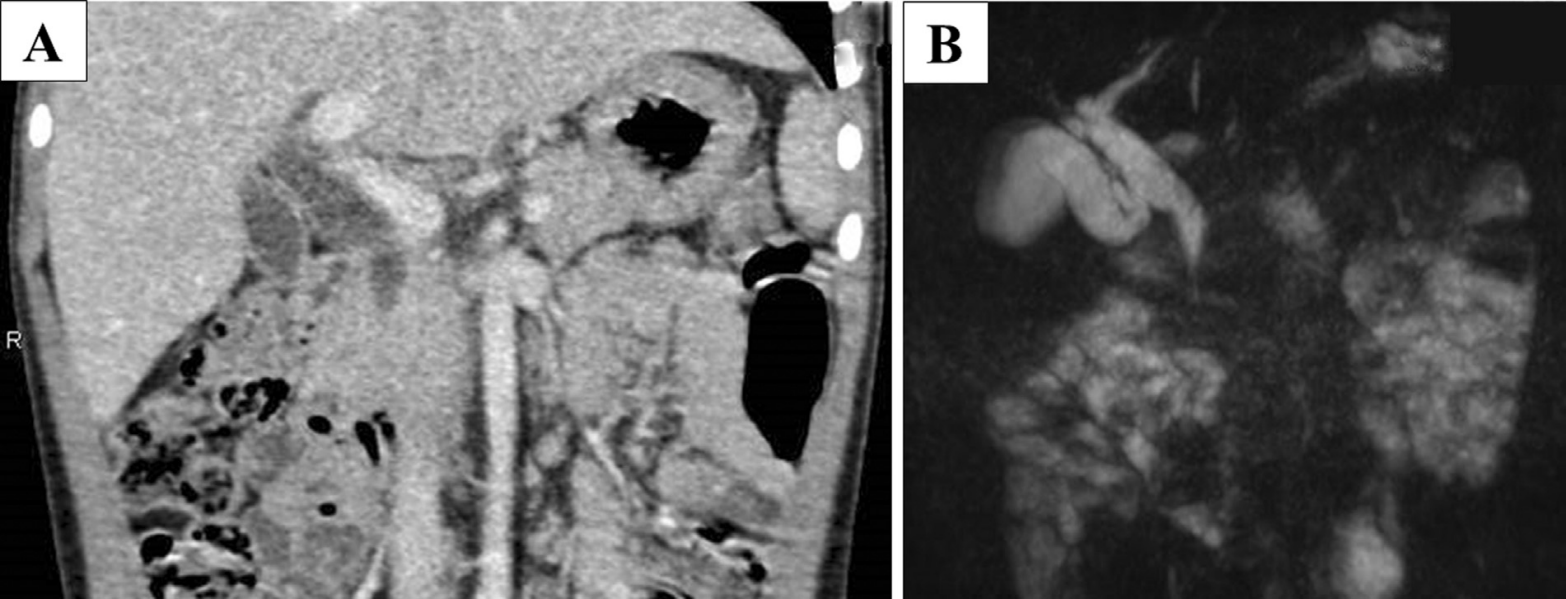
Computed tomography ( **A** ) and magnetic resonance cholangiopancreatography ( **B** ) show a 17-mm type Ia choledochal cyst according to the Todani classification.

A 4-year-old girl presented with abdominal pain and intermittent vomiting. Enhanced computed tomography (CT) and magnetic resonance cholangiopancreatography (MRCP) showed a 17-mm type Ia choledochal cyst according to the Todani classification ( Fig. 1A , 1B ). Definitive PBMJ was not detected based on CT and MRCP preoperatively.

Laparoscopic choledochal cyst excision was performed using five ports. Under general anesthesia, the patient was placed in a broad base position, and a 10-mm 30° laparoscope was inserted through an umbilical incision along with a trocar with a multichannel port device (E‧Z Access/LAP-PROTECTOR minimini; Hakko Co., Ltd., Tokyo, Japan). Pneumoperitoneum was established with 8-mm Hg CO _2_ insufflation. Three additional trocars and a 2.4-mm needle-type grasper (Teleflex, Morrisville, NC, USA) were inserted into the right upper abdomen (operator's left hand, 3.5 mm) and at the right side of the umbilicus (operator's right hand, 5 mm), the left lateral abdomen (assistant's left hand, 3.5 mm), and left upper abdomen (assistant's right hand, 2.4 mm).

**Fig. 2:**
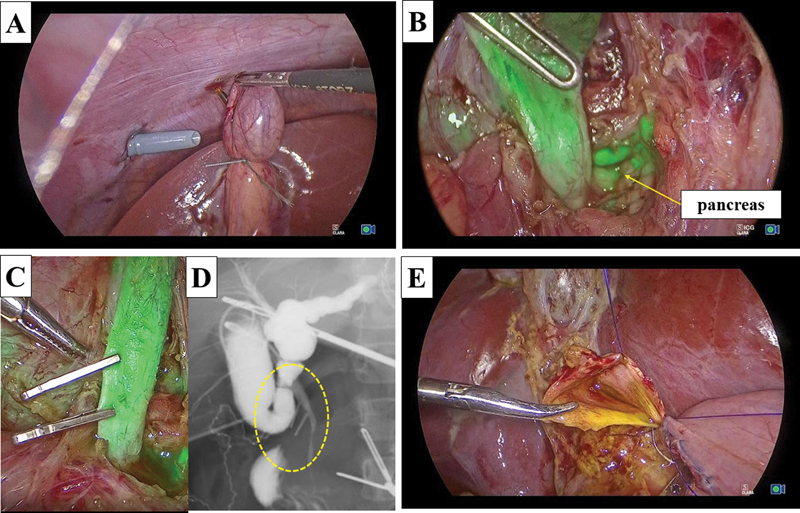
( **A** ) A mix of bile juice and ICG is co-injected into the gallbladder through a catheter. ( **B** ) Detection of the distal side of the cyst inside the pancreatic tissue to prevent injury of the pancreatic duct and tissue. ( **C** ) Application of metal clips just above the junction based on the findings of ICG imaging and cholangiography. ( **D** ) Confirmation of PBMJ (inside yellow circle) by intraoperative cholangiography using iodine contrast agent. ( **E** ) Approximation of both the posterior and anterior walls using interrupted intracorporal knot-tying with 5–0 absorbable sutures.

After dissecting the dilated common bile duct, a percutaneous silicon catheter was inserted into the gallbladder, and bile juice was aspirated. The amylase level of the bile juice was over 3 × 10 ^5^ IU/L, and PBMJ was suspected. Bile juice and ICG (Diagnogreen; Daiichi Sankyo, Tokyo, Japan) were mixed with the same concentration of ex vivo experiment and co-injected into the gallbladder through the catheter ( Fig. 2A ). The dilated common bile duct and pancreas were detected by NIR fluorescence imaging using a near-infrared/ICG camera (KARL STORZ). This imaging technique was useful for detecting the dissection margin of the distal side of the choledochal cyst inside the pancreatic tissue and preventing injury of the pancreatic tissue ( Fig. 2B ). Injury of the pancreatic duct and pancreas would be recognized as leakage of fluorescence fluid macroscopically if such an injury had occurred.

Because this was our first case using this technique, we also confirmed PBMJ with intraoperative cholangiography using iodine contrast agents. The distal side of the choledochal cyst was transected safely just above the junction based on the findings of ICG imaging and cholangiography ( Fig. 2C , 2D ). The jejunum was then extracted from the umbilical wound, and Roux-en Y jejunojejunostomy was performed. The mucosa and serosa of the opened hole was approximated using 6–0 absorbable sutures to secure hepaticojejunostomy. The jejunum was pulled up through the retro-colic. Both the posterior and anterior walls were approximated using interrupted intracorporeal knot-tying with 5–0 absorbable sutures ( Fig. 2E ). Anastomosis was completely performed without stent insertion.

The postoperative course was uneventful, with no complications reported. She was discharged on postoperative day 13. No complications occurred 4 months after the operation.

## Discussion

We have reported several techniques for laparoscopic choledochal cyst excision and hepaticojejunostomy in pediatric patients. 5 6 7 8 9 In our institution, some techniques have been standardized: (1) We enlarge the small hepatic duct using a diagonal cut up the left side of the hepatic duct, (2) the anastomotic hole is made at the anterior wall of the jejunum based on the size of the hepatic duct, and (3) the mucosa and serosa of the opened hole are approximated to perform membrane-to-membrane anastomosis. These tips are very useful for confirming the lumen of the hepatic duct. 5 However, the way to determine the transection level of a choledochal cyst has not yet been standardized, especially in non-dilated cases with PBMJ, because of the difficulty of confirming the junction in small patients. Repeatable intraoperative cholangiography and choledochoscopy are sometimes required for these cases.

Only one case of ICG fluorescence imaging in an adult choledochal cyst excision using the da Vinci system has been reported. 10 We herein report the first case of the performance of laparoscopic choledochal cyst excision and hepaticojejunostomy in a pediatric patient. Hirayama et al reported NIR fluorescence cholangiography with ICG for biliary atresia (BA) during the Kasai procedure. 11 ICG should be injected intravenously for BA patients 24 hours before the Kasai procedure. However, we were able to obtain a fluorescence image of the biliary tract by co-injection of bile and ICG just prior to the observation using the present technique.

Regarding the long-term complications derived from the remnant of the cyst, the development of malignancy following choledochal cyst excision has been reported. Cholangiocarcinoma occurs at various locations, including the remnant of the cyst and intrapancreatic duct. 12 To prevent cholangiocarcinoma during long-term follow-ups, complete excision of the choledochal cyst without a remnant duct is essential. We usually perform intraoperative cholangiography to determine the transection level of the pancreatic side of the choledochal cyst to ensure complete cyst excision. However, repeatable intraoperative cholangiography extends the operation time and increases the risk of radiation exposure. In the present case, we were unfortunately unable to detect the pancreatic duct in the pancreas with ICG fluorescence imaging because the ICG quickly spreads through the pancreatic tissue. By improving and modifying our technique, the pancreaticobiliary junction will be able to be visualized, and complete choledochal cyst excision will become feasible without extending the operation time or increasing the risk of radiation exposure.

ICG fluorescence imaging has been described as ICG combined with protein-generated fluorescent light. 4 In the preliminary study, ICG was mixed with bile ex vivo at various concentrations. We found that ICG requires at least the same amount of bile, and the 30% ICG concentration was deemed tentatively appropriate for visualizing fluorescent images. Allergy of ICG is extremely rare and there is no toxicity, 13 so co-injection of bile and ICG into the bile duct is deemed safe and feasible for detecting the bile duct intraoperatively. Further studies are needed to evaluate the best ratio of ICG and bile.

We report for the first time a novel technique for detecting the distal side of a choledochal cyst and possible PBMJ via the co-injection of bile and ICG in laparoscopic choledochal cyst excision and hepaticojejunostomy for a pediatric patient. This technique is very useful and can help determine the transection level of the distal side of the choledochal cyst without radiography.

## Notes

New Insights and the Importance for the Pediatric Surgeon Co-injection of bile and indocyanine green for detecting pancreaticobiliary maljunction of choledochal cyst is safe and low-invasive method without a risk of radiography and residual bile duct.
